# Does Activating the Human Identity Improve Health-Related Behaviors During COVID-19?: A Social Identity Approach

**DOI:** 10.3389/fpsyg.2022.810805

**Published:** 2022-03-23

**Authors:** David J. Sparkman, Kalei Kleive, Emerson Ngu

**Affiliations:** Department of Psychology, University of Wisconsin–Eau Claire, Eau Claire, WI, United States

**Keywords:** identification with all humanity (IWAH), social identity, COVID-19, health behaviors, helping

## Abstract

Taking a social identity approach to health behaviors, this research examines whether experimentally “activating” the human identity is an effective public-health strategy to curb the spread of COVID-19. Three goals of the research include examining: (1) whether the human identity can be situationally activated using an experimental manipulation, (2) whether activating the human identity causally increases behavioral intentions to protect the self and others from COVID-19, and (3) whether activating the human identity causally increases behaviors that help protect vulnerable communities from COVID-19. Across two preregistered experiments (total *N* = 675), results suggest (1) the manipulation of identification with humanity had a significant but small effect on participants’ psychological bond with all humanity (Cohen’s *d*s = 0.21 – 0.27), but not their concern for all humanity. However, the manipulation had (2) no causal effect on health-related behavioral intentions or (3) helping behaviors that reduce the spread of COVID-19. Limitations, future directions, and direct benefits of the research are discussed.

*“Seen from space, the Earth has no borders. The spread of the coronavirus is showing us that what we share is much more powerful than what keeps us apart. All people are inescapably interconnected, and the more we can come together to solve our problems, the better off we will all be.”*

– Scott Kelly.

## Introduction

In a New York Times opinion piece, astronaut Kelly (2020) provided a perspective on the interconnectedness among people at the beginning of a global pandemic. Included in this perspective is a possible remedy—not only to improve individual health and well-being—but also to improve our collective health and well-being. But as much research on the link between social psychological processes and health has shown, it is not simply the perception of *social connectedness* that affects health and well-being, but a shared *social identity* (Jetten et al., 2014).

According to social identity theory (SIT; Tajfel and Turner, 1979), a fundamental feature of the human experience centers around the psychological connection we have, and the meaning associated with, our shared (vs. unshared) social groups (e.g., gender identity, racial identity, nationality; also see Turner et al., 1987). According to self-categorization theory (SCT; Turner et al., 1987; Oakes et al., 1994), an extension of SIT, we have a range of social identities that exist at different levels of abstraction—that is, the individual level vs. social level vs. human level—all of which can be activated according to various personal and contextual factors. These complementary theories, now recognized as the social identity approach, were initially an explanation for intergroup attitudes and behavior (e.g., prejudice, discrimination, cooperation), but new research has shown social identities also have a strong impact on individual health and well-being because of the close connection between the self and the group (for reviews, see Haslam et al., 2009; Jetten et al., 2017). For instance, just during the COVID-19 pandemic alone, recent data from 67 countries suggest stronger identification with one’s nation predicted more positive well-being (Bonetto et al., 2021) and stronger adherence to recommended health behaviors (Van Bavel and Boggio, 2020). Despite an emerging research agenda that has wielded social identities as a tool for improving individual health and well-being (Jetten et al., 2017), comparatively little research has examined the impact of broader, more inclusive social identifications (e.g., human identity, global identity) on personal health behaviors that have implications for collective health. Thus, we argue that social identification at the most inclusive level of abstraction—the human level (Turner et al., 1987)—may be particularly well-suited to mobilize the kind of personal, health-related behaviors needed during a global pandemic.

### A Social Identity Approach to Health-Related Behaviors During COVID-19

Identification with humanity is uniquely suited for understanding responses to the COVID-19 outbreak because it is a global pandemic that cuts across national and cultural boundaries. COVID-19 is a collective crisis (rather than a crisis of the individual) that has affected nearly all human beings and revealed that we share a common fate (i.e., how the individual behaves has implications for spreading COVID-19 to others, and how others behave have implications for contracting COVID-19 for the self). Indeed, researchers have argued that social identification processes specific to the *human identity* become especially relevant “when an ingroup needs help fulfilling perceived basic human needs” (Carmona et al., 2020, p. 15), such as reducing illness or death among fellow humans. Drawing from the social identity model of pro-environmental action (SIMPEA; see Fritsche et al., 2018), appraising the COVID-19 pandemic as a collective threat should engender emotions and/or motivations that facilitate social identification processes, which in turn should facilitate behavioral responses that protect the self and others from COVID-19. To outline this process more specifically, an appraisal of the COVID-19 pandemic as a collective threat may engender motivations to reduce subjective uncertainty (Hogg, 2007), reduce the likelihood of existential mortality (Castano et al., 2002), or increase a sense of personal control (Fritsche et al., 2013). These motives, in turn, should lead to several interactive social identification processes, including ingroup identification, ingroup norms, and perceived efficacy of ingroup goals.

Identification with the human ingroup manifests as affective feelings of closeness and concern for the well-being of other humans—above and beyond the self (McFarland et al., 2012). In the context of COVID-19, human identification could manifest as a perceptual focus on the biological and sociocultural characteristics that all humans share—similarities that may be contrasted with the characteristics of non-human “outgroups” (Turner et al., 1987), such as the coronavirus itself (also see Reese et al., 2020). Perceived norms of the human ingroup during COVID-19 reflect an awareness of the agreed upon standards and prototypical patterns of behavior (e.g., mask wearing, social distancing) that serve to protect others from infection, whereas perceived efficacy reflects a belief in the likelihood that such ingroup norms will be effective against COVID-19. To the extent that these social identification processes become salient and internalized by the self, according to the SIMPEA approach, it should mobilize behaviors that protect the self and others from COVID-19 (e.g., wearing a mask, social distancing, sanitizing, vaccinating; see Fritsche et al., 2013; Reese et al., 2020). While these core social identification processes—ingroup identification, ingroup norms, and perceived efficacy of ingroup goals—are all important for understanding behavioral responses and appraisals during the COVID-19 pandemic, the primary focus of the current research is the link between identification with the human ingroup and its effect on personal, health-related behaviors.

Identification at the human level should be uniquely suited to promote personal behaviors that protect fellow humans from a viral outbreak, but very little research has addressed the link between identification with humanity and health-related behaviors during COVID-19. Moreover, the human identity (as opposed to the individual or social identity) is arguably the least researched level of abstraction in the social identity approach (e.g., see Wohl and Branscombe, 2005; for an updated review, see McFarland et al., 2019). Thus far, only a few studies have demonstrated that identification with all humanity (or global identification) uniquely predicts personal, health-related behaviors that reduce the spread of COVID-19 (Barragan et al., 2021; Wang et al., 2021; for helping behaviors during COVID-19, see Deng, 2021; Zagefka, 2021). However, these studies thus far have taken a correlational approach, operationalizing identification with all humanity as an individual difference measure rather than a social identity that is situationally activated. With this point in mind, only a few published studies have directly manipulated identification at the human level; of the studies that have, all examine its effect on improving intergroup relations or increasing international helping (Wohl and Branscombe, 2005; Greenaway et al., 2011; Reese et al., 2015). No research (to our knowledge) has attempted to experimentally manipulate identification with humanity with the goal of examining its causal effect on health-related COVID-19 behaviors (e.g., mask wearing, social distancing).

Overall, the importance of activating the human level of inclusiveness has not yet been fully realized as a potential public-health strategy for improving health-related behaviors during the COVID-19 pandemic. We argue that situationally activating identification with the human ingroup should increase one’s psychological bond with and concern for the well-being of fellow humans. This focus on others (rather than the self) should promote health-related behaviors (e.g., mask wearing, social distancing) that benefit and protect the human ingroup from contracting COVID-19. Therefore, the current research will test the public-health utility of the human identity by directly “activating” it using an experimental manipulation, and examining whether it causally promotes personal, health-related behaviors needed to reduce the spread of COVID-19.

### The Present Research

The present research takes an important experimental approach with three primary goals**. Goal 1**: Examine whether the human identity can be situationally activated using random assignment to a control vs. experimental condition. Because only a few published studies have shown success in directly manipulating the human identity, doing so remains relatively uncharted experimental territory. Indeed, recent research involving 11 experiments with different manipulations (e.g., educational activities, persuasion, subliminal priming, self-awareness) have demonstrated the difficulty in causally increasing social identification at the highest, most inclusive level of abstraction (see Reysen et al., 2021). Given this uncertainty, in Study 1 we tested the effectiveness of two different manipulations of the human identity in the hopes that at least one would increase identification with humanity. **Goal 2**: Examine whether activating the human identity causally increases intentions to engage in behaviors that protect the self and others from COVID-19, including wearing a mask, cleaning, social distancing, and receiving a COVID-19 “booster” vaccine in the future. **Goal 3**: Examine whether activating the human identity causally increases actual behaviors geared toward helping vulnerable communities become more protected from COVID-19.

### Statement on Open Science

To increase transparency in psychological science, the hypotheses, manipulations and measures, sampling plan, data exclusion plan, and data analytic plan of this research were preregistered on the Open Science Framework (for Study 1^1^, for Study 2^2^). Although preregistrations were submitted after data collection began, no summary statistics or analyses were conducted until data collection was complete. A copy of the data (including raw data files, SPSS data files, and SPSS syntax for variable creation and primary analyses) and survey materials can be found on OSF^3^.

## Study 1

### Method

#### Sample Size and Power Analyses

Using G*Power software (Faul et al., 2007) and the ANCOVA statistical test (results were also similar for an ANOVA), a preregistered, *a priori* power analysis was conducted to estimate required sample size. To date, no research has examined the effect of a manipulation of identification with humanity on health-related behaviors. However, prior research similar to the current study (see Reese et al., 2015) has shown that manipulating identification with humanity increases the behavioral component of human identification (*d* = 0.41) and increases donations to a local charity (*d* = 0.43). To achieve at least 80% statistical power with input parameters of a small-to-medium effect size (Cohen’s *d* = 0.41, Cohen’s *f* = 0.205), conventional significance level (*p* < .05), two numerator degrees of freedom, three conditions, and two covariates, the required total sample size was at least 233 participants. To further increase the power of the study, a total sample size of at least 300 participants was set. After applying exclusion criteria, 297 participants were available for primary analyses (see below section). Based on the results of a sensitivity power analysis using the above criteria, this final sample (*N* = 297) yielded a required effect size of Cohen’s *d* = 0.36 (Cohen’s *f* = 0.181).

#### Participants and Procedure

From November 20, 2020, to April 18, 2021, a total of 302 participants were recruited from an undergraduate psychology subject pool at a mid-size university in the state of Wisconsin, United States. It is worth noting that this university held in-person classes throughout most of the 2020–2021 academic year, though masks were required in classrooms, desks were socially distanced, and student symptoms were monitored via a smartphone application. Consistent with pre-registered data exclusion criteria, four participants were removed for not having enough responses to compute an average on one or more measures, and an additional participant was removed for not completing the manipulation. This left a final sample size of 297 participants for analyses. Demographically, participants were primarily White (86.9% White, 1.3% Black/African-American, 2% Hispanic/Latino, 3.4% Asian/Pacific Islander, 5.6% Biracial/Multiracial, 0.7% Other), primarily women (79.5% women, 18.5% men, 2% other), and ranged in age from 18 to 52 years old (*M* = 19.96 years old, *SD* = 3.15).

Participants could complete our study in exchange for satisfying a course requirement or earning extra credit toward a course. The study was described as an exploration of the visual, emotional, and personality correlates of cognitive intelligence. A second portion of the study, which was described as separate from examining the correlates of cognitive intelligence, was to ‘‘explore people’s opinions and behaviors regarding current COVID-19 guidelines.’’ However, these separate studies were connected, with COVID-19 behaviors representing the primary dependent variables (see below). After providing informed consent, participants completed measures of empathy, personality, and three multiplication questions to buttress the cover story. Participants were then randomly assigned to one of three conditions manipulating identification with humanity. Next, participants completed a manipulation check, primary dependent measures of behavioral intentions to minimize the spread of COVID-19 and an optional charity donation task, followed by demographic questions.^4^ Participants were debriefed at a later date.

#### Manipulating Identification With Humanity

To buttress the cover story of the research, participants were told a “simple test” was developed to measure their visual memory capacity and language ability. Participants were asked to carefully view an image for 30 s and try to remember as many of its details as possible, with a visible, 30-s timer displayed on the page. To “measure visual memory capacity,” we then asked participants three questions about the image they viewed. To “measure their language ability,” we asked participants to take 3 min to type a response to a “conceptual question.” A visible timer was set for 3 min, and we set a minimum requirement of 150 characters for the writing prompt. In both cases, participants could not advance to the next page until the timers expired.

Participants randomly assigned to the control condition (*n* = 101) viewed a house-like image composed of different shapes (e.g., a square, parallelogram, triangle). Their writing prompt asked, “What does it mean to be considered a triangle? And how is this similar to, or different from, what it means to be a rectangle?” Participants randomly assigned to the first experimental condition viewed a realistic picture of the earth from space (*n* = 98, the “earth condition”), and those assigned to the second experimental condition viewed an animated picture of the earth in space surrounded by human hands (*n* = 98, the “globe condition,” used in prior research; see Reese et al., 2015). The writing prompt, which was based on a colleagues’ unpublished data (Bertin, 2019) was used in both experimental conditions and asked, “What does it mean to you to be a human being? What do you have in common with, or in what ways are you connected to, humans all over the world?” Considering the effect of social identity on health-related behavior depends on whether the identity is salient and meaningful to the self (Jetten et al., 2017), this manipulation makes the human identity salient and then asks participants to reflect on its personal meaning to them.

#### Manipulation Check

As a check on the effectiveness of the manipulation, we used the *identification with all humanity* (α = .82) scale from prior research^5^ (McFarland et al., 2012). Based on the results of a factor analysis and consistent with other findings (Reese et al., 2015; Reysen and Hackett, 2016; McFarland et al., 2019; Hamer et al., 2021), this scale broke down into two subfactors that researchers have increasingly termed *bond with all humanity* (α = .76) and *concern for all humanity* (α = .75). These two subfactors represent the identity and behavioral components of identification with all humanity, respectively (see Hamer et al., 2021). Identification with one’s community and other Americans were also measured but not used in the present research.

#### Health-Related Behavioral Intentions

For the purposes of this research, we constructed a detailed series of questions measuring participants’ intentions to wear a mask, sanitize, and social distance across different contexts and situations (e.g., indoor vs. outdoor settings, public vs. private settings). We believed this approach would be more realistic and have greater reliability than asking for single-item questions about intentions to wear a mask, sanitize, and social distance in general.

Prior to answering any questions of their behavioral intentions, participants were reminded that they were now completing “a separate part of this study.” Ten questions measured intentions to *wear a mask* (α = .87; e.g., “Imagine you visit an indoor private gathering, such as inside the home of friends or family who are not current household members: do you intend to regularly wear a mask?” and “Imagine you visit an indoor public place, such as a restaurant, bar, grocery store, library, or shop: do you intend to regularly wear a mask?”). Twelve questions measured intentions to *clean, wash, or sanitize* (α = .91; e.g., “Do you intend to sanitize frequently used surfaces in public spaces after each use, such as tables, gym equipment, computer equipment, and gas pumps?” and “Do you intend to clean your hands (with hand sanitizer or soap and water) after touching frequently used surfaces?”). Finally, ten questions measured intentions to *social distance* (α = .88; e.g., “Do you intend to regularly maintain a six-foot distance from people who are not current household members?” and “Imagine you visit an indoor public space, such as a restaurant, bar, grocery store, library, or shop: do you intend to regularly maintain a six-foot distance from other people?” [reverse-scored]). All questions were framed as intentions to engage in these behaviors *in the next week*, and all questions were answered using the same 1 (*very unlikely*) to 6 (*very likely*) scale. Although our preregistration committed us to measuring and analyzing these behaviors separately, the mask wearing, cleanliness, and social distancing measures were strongly correlated (*r*s ranged from .56 to .79, *p* < .001) and could be combined to form an overall composite of *health-related behavioral intentions* (α = .94).

#### Helping Behavior

As a measure of actual behavior, participants could learn how to help raise money (up to $100 donated by the researchers) for the #MaskUpMKE fund of the United Way of Greater Milwaukee and Waukesha County, an initiative to make and distribute face masks to essential workers, communities of color, and other individuals in the region. It was made clear that participants were “under no obligation to participate, and choosing not to participate will in no way affect your ability to earn your credit.” Thus, participants’ decision to “finish the study and earn my SONA credit” or “learn how to help the #MaskUpMKE fund” represented our dichotomous measure of *helping interest* (0 = no, 1 = yes). Participants who chose to finish the study without helping were directed to the final page of the survey, whereas participants who chose to learn more about the helping opportunity were sent to a separate page.

Participants who opted to help with the donation opportunity were told, “the researchers… have developed a simple way to quantify how much effort people are willing to give to help contribute to the #MaskUpMKE fund.” Using a list of 100 randomly generated strings of nine-digit numbers, participants were asked to identify whether a total of 20 number strings were included on a primary list. For every number correctly identified from the primary list, $0.05 would be donated to the #MaskUpMKE fund. However, if a number was incorrectly identified from the primary list, $0.05 would not be added to the participant’s donation amount. Participants were reminded that the task is voluntary, and that they can complete “as few or as many number searches” as they would like. The amount of donation money participants secured by correctly identifying number strings represented our continuous measure of *helping effort*, which ranged from $0.00 to $1.00. Those who opted out of the task received a score of $0.00.

#### Covariates

Considering data collection for this study was slow and progressed over the course of 6 months, we wanted to control for the possible effects of time and variability in COVID-19 cases on our primary dependent variables. We suspected that as time progressed and COVID-19 cases slowly decreased into the spring of 2021, participants would find it increasingly less important to engage in behaviors that minimize the spread of COVID-19. As outlined in our preregistration materials, a measure of *time* was computed by determining the day participants completed the study (range: Day 1 – Day 150), and a measure of *COVID-19 positivity rates* for the county were taken from the CDC’s county-level time series data for the state of Wisconsin. Positive test data are reported by the CDC as weekly averages by day, which were matched with participants’ study completion date.

#### Preregistered Hypotheses

We hypothesized that, compared to the control condition, participants in the “earth” condition would report stronger (1) identification with all humanity (as measured by the full scale; *Hypothesis 1a*), (2) bond with all humanity factor (*Hypothesis 1b*), and (3) concern for all humanity factor (*Hypothesis 1c*). We had the same hypotheses when comparing the control to the “globe” identification with humanity condition (*Hypotheses 2a – 2c*). Controlling for time and COVID-19 positivity rates, we also hypothesized that, compared to the control condition, participants in the “earth” identification with humanity condition would (1) report stronger intentions to wear a mask (*Hypothesis 3a*), clean, wash, or sanitize (*Hypothesis 3b*); and social distance (*Hypothesis 3c*); (2) be more likely to express interest in learning how they can help secure masks for others (*Hypothesis 3d*), and (3) put more effort toward a task that contributes money to help secure masks for others (*Hypothesis 3e*). We had the same hypotheses when comparing the control to the “globe” identification with humanity condition (*Hypotheses 4a – 4e*). Finally, we remained agnostic and made no specific hypotheses regarding differences between the two experimental conditions.

#### Analytic Strategy

Consistent with our preregistration, separate one-way analyses of variance (ANOVA) tested whether the manipulation of identification with humanity was successful in affecting the full scale of identification with all humanity, the bond with all humanity factor, and concern for all humanity factor. Next, after controlling for time and COVID-19 positivity rates, separate one-way analyses of covariance (ANCOVA) tested whether the manipulation of identification with humanity affected behavioral intentions to wear a mask, clean, and social distance, as well as how much effort participants put toward helping others secure masks. Where appropriate, significant ANOVAs and ANCOVAs were interpreted with *post hoc* pairwise comparisons using the least-significant difference (LSD) test. Finally, after controlling for time and COVID-19 positivity rates, a binary logistic regression tested whether the manipulation of identification with humanity affected interest in helping others secure masks (yes or no).

### Results

For descriptive statistics and bivariate correlations of all primary measured variables, see Table 1.

**TABLE 1:** Descriptive statistics and bivariate correlations of all primary measured variables in Study 1.

Variable	*M (SD)*	1	2	3	4	5	6	7	8	9	10
(1) IWAH Full Scale	3.30 (0.63)	–									
(2) IWAH Bond	2.72 (0.74)	.90***	–								
(3) IWAH Concern	4.01 (0.70)	.83***	.50***	–							
(4) Mask Wearing	4.30 (1.06)	.23**	.20***	.19***	–						
(5) Cleanliness	4.48 (1.06)	.36***	.30***	.33***	.59***	–					
(6) Social Distancing	3.98 (1.12)	.23***	.18**	.22***	.79***	.56***	–				
(7) Health Composite	4.27 (0.94)	.32***	.27***	.29***	.90***	.84***	.89***	–			
(8) Helping Interest	NA	.11*	0.06	.14*	.09	.12*	.13*	.13*	–		
(9) Helping Effort	0.05 (0.19)	.10*	.08	.11*	.10*	.10*	.19**	.15*	.81***	–	
(10) Time	NA	–.07	–.04	–.09	−.18**	−.17**	−.24***	−.22***	–.02	–.04	–
(11) COVID Rates	7.74 (4.07)	.10*	.08	.10*	.17**	.18**	.24***	.23***	.01	.04	−.95***

***p < .001, **p < .01, *p < .05, ^†^p < .09. IWAH: Identification with All Humanity. Health Composite: the average of all mask wearing, cleanliness, and social distancing items. Helping interest (0 = no, 1 = yes) does not have descriptive statistics because it is a dichotomous variable, and the covariate of Time ranges from 1 to 150 days.

#### Manipulation Check

Regarding Hypotheses 1a – 2c, the manipulation of identification with humanity had no effect on the full scale of identification with all humanity, omnibus *F*(2, 294) = 0.73, η_*p*_^2^ = 0.01, *p* = .49; the bond with all humanity factor, omnibus *F*(2, 294) = 1.88, η_*p*_^2^ = 0.01, *p* = .16; nor the concern for all humanity factor, omnibus *F*(2, 294) = 0.02, η_*p*_^2^ ≤ 0.001, *p* = .98 (for descriptive and additional inferential statistics, including effect sizes, see Table 2). Although all omnibus tests were not significant, examining the means and exploring the results of pairwise comparisons revealed that bond with all humanity was marginally significantly higher in the “earth” identification with humanity condition (*M* = 2.80) compared to the control (*M* = 2.61), *p* = .07. This provides some support for Hypothesis 1b, although we interpret this with caution. All other pairwise comparisons were non-significant, including the full identification with all humanity scale, *p*s > .27; and the concern for all humanity factor, *p*s > .86.

**TABLE 2:** Descriptive statistics and pairwise comparison results of ANOVAs, ANCOVAs, and binary logistic regression in Study 1.

Variable	(1) Control *M* (*SD*)	(2) Human ID “Earth” *M* (*SD*)				95% C.I.	
		(3) Human ID “Globe” *M* (*SD*)		*d*	*p*	Lower	Upper
IWAH^a^ (Full scale)	(1) 3.23 (0.53)	(2) 3.33 (0.65)		0.17	.27	–0.27	0.08
		(3) 3.32 (0.69)		0.15	.33	–0.26	0.09
Bond^a^ (Subscale)	(1) 2.61 (0.64)	(2) 2.80 (0.76)		0.27	.07	–0.40	0.02
		(3) 2.76 (0.80)		0.21	.14	–0.36	0.05
Concern^a^ (Subscale)	(1) 4.01 (0.66)	(2) 4.00 (0.69)		0.01	.86	–0.18	0.22
		(3) 4.02 (0.77)		0.01	.98	–0.20	0.19
Mask wearing^b^	(1) 4.33 (1.09)	(2) 4.27 (1.09)		0.06	.70	–0.24	0.35
		(3) 4.29 (1.01)		0.04	.81	–0.26	0.33
Cleaning^b^	(1) 4.52 (1.03)	(2) 4.49 (1.11)		0.03	.85	–0.27	0.32
		(3) 4.45 (1.04)		0.07	.68	–0.23	0.36
Social distancing^b^	(1) 4.06 (1.14)	(2) 3.94 (1.15)		0.10	.47	–0.19	0.42
		(3) 3.95 (1.07)		0.10	.53	–0.21	0.40
Health composite^b^	(1) 4.31 (0.95)	(2) 4.25 (0.99)		0.06	.63	–0.19	0.32
		(3) 4.24 (0.88)		0.08	.62	–0.19	0.32
Helping effort^b^	(1) .05 (0.21)	(2) .06 (0.22)		0.05	.90	–0.06	0.05
		(3) .03 (0.14)		0.11	.48	–0.04	0.07
						95% C.I. *OR*	
		*b* (*SE*)	Wald	*OR*	*p*	Lower	Upper

Helping interest^c^	(1) (Reference)	(2) –0.09 (0.51)	0.03	0.91	.85	0.34	2.47
		(3) –0.25 (0.53)	0.22	0.78	.64	0.28	2.19

*Superscript^a^ indicates effects were tested using an ANOVA, superscript^b^ indicates effects were tested using an ANCOVA (controlling for time and COVID-19 positivity rates), and superscript^c^ indicates effects were tested using a binary logistic regression (controlling for time and COVID-19 positivity rates). Adjusted means and standard errors for ANCOVAs are not reported because covariates were not significant. The progression of time was unrelated to mask wearing, F(1,292) = 0.64, η_p_^2^ = 0.002, p = .42; cleaning, F(1,292) = 0.05, η_p_^2^ ≤ 0.001, p = .82; social distancing, F(1,292) = 0.13, η_p_^2^ ≤ 0.001, p = .72; the overall composite of health-related behavioral intentions, F(1,292) = 0.26, η_p_^2^ ≤ 0.001, p = .61; and helping effort, F(1,292) = 0.05, η_p_^2^ ≤ 0.001, p = .83. COVID-19 positivity rates was unrelated to mask wearing, F(1,292) = 0.05, η_p_^2^ ≤ 0.001, p = .82; cleaning, F(1,292) = 0.61, η_p_^2^ = 0.002, p = .44; social distancing, F(1,292) = 1.12, η_p_^2^ = 0.004, p = .29; the overall composite of health-related behavioral intentions, F(1,292) = 0.65, η_p_^2^ ≤ 0.002, p = .42; and helping effort, F(1,292) = 0.20, η_p_^2^ = 0.001, p = .66. Time was unrelated to helping interest, b = –0.01, SE = 0.01, Wald = 0.43, OR = 0.99, 95% CI of OR [0.97, 1.02], p = .51; and COVID-19 positivity rates was unrelated to helping interest, b = –0.09, SE = 0.16, Wald = 0.32, OR = 0.91, 95% CI of OR [0.66, 1.25], p = .57.*

#### Health-Related Behavioral Intentions

Regarding Hypotheses 3a – 3c and Hypotheses 4a – 4c, the manipulation of identification with humanity (even after controlling for time and COVID-19 positivity rates) had no effect on intentions to wear a mask, omnibus *F*(2, 292) = 0.08, η_*p*_^2^ = 0.001, *p* = .93; intentions to clean, wash, or sanitize, omnibus *F*(2, 292) = 0.09, η_*p*_^2^ = 0.001, *p* = .92; or intentions to social distance, omnibus *F*(2, 292) = 0.32, η_*p*_^2^ = 0.002, *p* = .73. We also looked at the effect of the manipulation of identification with humanity on the overall composite of health-related behavioral intentions, which remained non-significant, omnibus *F*(2, 292) = 0.16, η_*p*_^2^ = 0.001, *p* = .85 (see Table 2).

#### Helping Behaviors

Regarding Hypotheses 3d and 4d, a model including the manipulation of identification with humanity, time, and COVID-19 positivity rates did not predict participants’ interest in learning how to help secure masks for others, omnibus χ^2^(4, *N* = 297) = 0.68, *p* = .95. Testing Hypotheses 3e and 4e, the manipulation of identification with humanity (even after controlling for time and COVID-19 positivity rates) had no effect on participants’ effort to help secure masks for others, omnibus *F*(2, 292) = 0.40, η_*p*_^2^ = 0.003, *p* = .67 (see Table 2).

### Discussion

Results suggest the manipulation of identification with humanity did not causally promote stronger intentions to wear a mask, clean, or social distance, nor greater interest in learning how to help or actual effort needed to help secure masks for others. Despite these non-significant effects on the primary dependent variables, preliminary findings indicate the manipulation of identification with humanity may be effective at activating this inclusive social identity. Compared to the control, the “earth” identification with humanity condition (but not the “globe” condition) marginally increased identification with all humanity. However, this increase appeared only for the bond with all humanity factor. To assess the reliability of the effect of the manipulation on bond with all humanity, and to again examine whether identification with humanity causally promotes health-related COVID-19 behaviors, we conducted a follow-up study with greater statistical power and a more diverse, non-student sample.

## Study 2

### Method

#### Sample Size and Power Analyses

Using G*Power software and the ANCOVA statistical test (results were similar for an independent-samples *t*-test), an *a priori* power analysis was conducted to estimate required sample size. To ensure the current study at least demonstrates a significant effect of the manipulation on identification with humanity, the marginally significant effect size for bond with all humanity was used from Study 1. To achieve 80% statistical power with input parameters of a small effect size (Cohen’s *d* = 0.27, Cohen’s *f* = 0.135), conventional significance level (*p* < .05), one numerator degree of freedom, two conditions, and one covariate, the required total sample size was at least 433 participants. Given a fixed availability of personal funds, however, we sought to recruit a total sample size as close to this number as possible. Therefore, our goal was a total sample size of 400 participants, which represents approximately 77% statistical power to discover a significant effect. After applying exclusion criteria, 378 participants were available for primary analyses (see below section). Based on the results of a sensitivity power analysis using the above criteria, this final sample (*N* = 378) yielded a required effect size of Cohen’s *d* = 0.29 (Cohen’s *f* = 0.144).

#### Participants and Procedure

On May 14, 2021, a total of 389 participants^6^ from across the United States were recruited on the Prolific crowdsourcing platform. Consistent with pre-registered data exclusion criteria, 10 participants were removed for having a relative survey completion speed twice as fast as the typical participant, and an additional participant was removed for not following instructions for the experimental manipulation. This left a final sample of 378 participants for analyses. Demographically, participants were primarily White (65.6% White, 8.5% Black/African-American, 5.3% Hispanic/Latino, 11.1% Asian/Pacific Islander, 0.8% Native American, 7.9% Biracial/Multiracial, 1.1% Other), primarily women (60.8% women, 36.5% men, 2.6% other), and ranged in age from 18 to 76 years old (*M* = 36.62 years old, *SD* = 13.18).

Participants could earn at least $1.56 in exchange for completing our study (completion time: *M* = 14.90 min, *SD* = 10.07)^7^, which was described on Prolific in the same way as Study 1 (i.e., a “two-part” study exploring the visual, emotional, and personality correlates of cognitive intelligence, and opinions and behaviors regarding current COVID-19 guidelines). After providing informed consent, participants completed measures of empathy, personality, and two multiplication questions to buttress the cover story. Participants were then randomly assigned to a control or experimental condition manipulating identification with humanity. Next, participants completed a manipulation check, primary dependent measures of behavioral intentions to minimize the spread of COVID-19 and an optional charity donation task, followed by demographic questions. Participants were debriefed at a later date.

#### Manipulating Identification With Humanity

The same manipulation of identification with humanity from Study 1 was used, but with three small modifications. Fist, when participants were instructed to view an image for the “visual memory capacity test,” a visible, 35-s timer (instead of 30 s) was displayed to provide more time for participants to get oriented to the image. Second, when participants completed the conceptual question measuring their “language ability,” a visible timer was set for 2 min and 35 s (instead of 3 min) to reduce survey completion time and minimize payment costs. Finally, participants were randomly assigned to the same control condition (*n* = 190) or “earth” identification with humanity condition (*n* = 188) from Study 1. Because the manipulation-check effect size from the “globe” experimental condition was slightly smaller than the “earth” condition, it was eliminated to increase sample size. All other features of the manipulation remained the same.

#### Manipulation Check

As a check on the effectiveness of the manipulation, the same *identification with all humanity* (α = .90) scale from Study 1 was used, including factors of *bond with all humanity* (α = .84) and *concern for all humanity* (α = .83). To reduce survey length, identification with one’s community and other Americans were not measured.

#### Health-Related Behavioral Intentions

As in Study 1, participants were reminded that they were now completing “a separate part of this study” prior to measuring their health-related behavioral intentions. Participants were told, “Many infectious disease experts are concerned that currently approved COVID-19 vaccinations will eventually be ineffective at stopping the spread of COVID-19, especially as the virus mutates over time. To slow the spread of COVID-19 across the world, infectious disease experts believe people (even those who are currently vaccinated) will still need to practice certain behaviors to slow the spread of COVID-19.” To reduce survey completion time, we asked participants their likelihood of engaging in only three behaviors that reduce the chance of contracting COVID-19 and spreading it to other people, including whether they would “seek out a COVID-19 ‘booster’ vaccination in the future,” “wear a mask in crowded public areas in the future,” and “practice social distancing in crowded public areas in the future.” Each question was answered on the same 0 (*very unlikely*) to 100 (*very likely*) scale. As in Study 1, our preregistration committed us to measuring and analyzing these behaviors separately, but the COVID-19 booster vaccination, mask wearing, and social distancing items could be combined to form an overall composite of *health-related behavioral intentions* (α = .76).

#### Helping Behavior

As a measure of actual behavior, participants could learn how to help raise money (up to $100 donated by the researchers) in the same way as Study 1, but with two modifications. Instead of helping secure masks for the #MaskUpMKE fund, participants could help raise money for the Vaccine Access Fund, an initiative providing free rides to vaccine sites for people in communities hit hard by the pandemic, including Black and Latino adults, people living on low incomes, and others for whom transportation is a barrier. Participants’ decision to “finish the study and earn my payment” or “learn how to help the Vaccine Access Fund” represented our dichotomous measure of *helping interest* (0 = no, 1 = yes). Those who opted to help with the donation opportunity completed the same number-search task as in Study 1, but instead of earning $0.05 for every correctly identified number string, participants could earn $0.20 for each correct number. Given the low level of participation from Study 1, the donation amount was increased to provide more incentive to participants. All other features of the number-search task remained the same. The amount of money participants secured during the number-search task represented our continuous measure of *helping effort*, which ranged from $0.00 to $4.00. Those who opted out of the task received a score of $0.00.

#### Covariate

Because the study was launched soon after the Centers for Disease Control suggested vaccinated individuals no longer needed to wear masks in most situations, we measured and controlled for participants’ *vaccination status* by asking, “Are you currently fully vaccinated by an approved COVID-19 vaccine?” (0 = no, 1 = yes). At the time of data collection, 53.2% of participants were fully vaccinated.

#### Preregistered Hypotheses

We hypothesized that, compared to the control condition, participants in the identification with humanity condition would report (1) stronger identification with all humanity (as measured by the full scale; *Hypothesis 1a*), (2) stronger bond with all humanity (*Hypothesis 1b*), and (3) no significant difference in concern for all humanity (*Hypothesis 1c*). Controlling for COVID-19 vaccination status, we also hypothesized that, compared to the control condition, participants in the identification with humanity condition would report stronger intentions to (1) receive a COVID-19 “booster” vaccination (*Hypothesis 2a*), wear a mask (*Hypothesis 2b*), and social distance (*Hypothesis 2c*); (2) be more likely to express interest in learning how they can help others secure rides to a vaccination appointment (*Hypothesis 2d*), and (3) put more effort toward a task that contributes money to help others secure rides to a vaccination appointment (*Hypothesis 2e*).

#### Analytic Strategy

Consistent with preregistration, separate independent-samples *t*-tests assessed whether the manipulation of identification with humanity affected identification with all humanity, the bond with all humanity factor, and the concern for all humanity factor. Next, after controlling for vaccinations status, separate one-way analyses of covariance (ANCOVA) tested whether the manipulation of identification with humanity affected behavioral intentions to receive a “booster” vaccination, wear a mask, and social distance, as well as how much effort participants put toward helping others secure rides to vaccination appointments. Finally, after controlling for vaccination status, a binary logistic regression tested whether the manipulation affected interest in helping others secure rides to vaccination appointments (yes or no).

### Results

For descriptive statistics and bivariate correlations of all primary measured variables, see Table 3.

**TABLE 3:** Descriptive statistics and bivariate correlations of all primary measured variables in Study 2.

Variable	*M (SD)*	1	2	3	4	5	6	7	8	9
(1) IWAH Full Scale	3.50 (0.75)	–								
(2) IWAH Bond	3.25 (0.83)	.95***	–							
(3) IWAH Concern	3.81 (0.79)	.90***	.71***	–						
(4) Booster Vaccine	73.46 (32.76)	.17**	.12*	.20***	–					
(5) Mask Wearing	83.22 (25.56)	.22***	.16**	.25***	.43***	–				
(6) Social Distancing	80.88 (24.52)	.18***	.15**	.19***	.44***	.78***	–			
(7) Health Composite	79.19 (22.91)	.23***	.17***	.26***	.79***	.85***	.86***	–		
(8) Helping Interest	NA	.10^†^	.05	.15**	.28***	.26***	.21***	.31***	–	
(9) Helping Effort	0.94 (1.50)	.08	.04	.10*	.24***	.19***	.15**	.24***	.75***	–
(10) Vaccine Status	NA	.01	–.02	.03	.44***	.20***	.14**	.34***	.19***	.16**

***p < .001, **p < .01, *p < .05, ^†^p < .06. IWAH: Identification with All Humanity. Health composite: the average of the booster vaccine, mask wearing, and social distancing items. Helping interest (0 = no, 1 = yes) and the covariate of vaccination status (0 = no, 1 = yes) do not have descriptive statistics because they are dichotomous variables.

#### Manipulation Check

Supporting Hypotheses 1a – 1c, the manipulation of identification with humanity (compared to the control condition) marginally increased the full scale of identification with all humanity, *t*(376) = –1.68, *p* = .09; significantly increased bond with all humanity, *t*(376) = –2.30, *p* = .02; but had no effect on concern for all humanity, *t*(376) = –0.58, *p* = .56 (for descriptive and additional inferential statistics, including effect sizes, see Table 4).

**TABLE 4:** Descriptive statistics and results of *t*-tests, ANCOVAs, and binary logistic regression in Study 2.

Variable	Control Condition *M* (*SD*)	Human ID Condition *M* (*SD*)				95% C.I.	
	[Adjusted *M* (*SE*)]	[Adjusted *M* (*SE*)]		*d*	*p*	Lower	Upper
IWAH^a^ (Full scale)	3.43 (0.73)	3.56 (0.77)		0.17	.09	–0.28	0.02
Bond^a^ (Subscale)	3.15 (0.82)	3.35 (0.84)		0.24	.02	–0.36	–0.03
Concern^a^ (Subscale)	3.78 (0.77)	3.83 (0.80)		0.06	.56	–0.21	0.11
“Booster” Vaccine^b^	75.53 (31.44) [74.49 (2.15)]	71.36 (34.00)		0.12	.50	–3.92	8.07
		[72.42 (2.16)]					
Mask wearing^b^	83.78 (24.14) [83.40 (1.82)]	82.65 (26.96)		0.04	.89	–4.72	5.46
		[83.03 (1.83)]					
Social distancing^b^	79.66 (24.38) [79.39 (1.76)]	82.12 (24.67)		0.10	.23	–7.92	1.93
		[82.39 (1.77)]					
Health composite^b^	79.66 (22.53) [79.09 (1.57)]	78.71 (23.33)		0.04	.93	–4.57	4.21
		[79.28 (1.58)]					
Helping effort^b^	0.90 (1.49) [0.88 (0.11)]	0.99 (1.51)		0.06	.42	–0.43	0.18
		[1.00 (0.11)]					
						95% C.I. *OR*	
		*b* (*SE*)	Wald	*OR*	*p*	Lower	Upper

Helping interest^c^	(Reference)	0.15 (0.21)	0.47	1.16	.49	0.76	1.76

*Superscript^a^ indicates effects were tested using a t-test, superscript^b^ indicates effects were tested using an ANCOVA (controlling for COVID-19 vaccination status), and superscript^c^ indicates effects were tested using a binary logistic regression (controlling for COVID-19 vaccination status). For ANCOVA results, unadjusted means and standard deviations are reported first, and adjusted means and standard errors are reported in brackets. Vaccination status was positively related to intentions to receive the “booster” vaccine, F(1,375) = 86.75, η_p_^2^ = 0.19, p < .001; intentions to wear a mask, F(1,375) = 16.07, η_p_^2^ = 0.04, p < .001; intentions to social distance, F(1,375) = 8.40, η_p_^2^ = 0.02, p < .01; the overall composite of health-related behavioral intentions, F(1,375) = 47.21, η_p_^2^ = 0.11, p < .001; and helping effort, F(1,375) = 9.68, η_p_^2^ = 0.03, p < .01. Vaccination status was also positively related to helping interest, b = 0.80, SE = 0.22, Wald = 13.66, OR = 2.22, 95% CI of OR [1.46, 3.39], p < .001.*

#### Health-Related Behavioral Intentions

Regarding Hypotheses 2a – 2c, the manipulation of identification with humanity (even after controlling for COVID-19 vaccination status) had no effect on intentions to receive a “booster” vaccine, *F*(1, 375) = 0.46, η_*p*_^2^ = 0.001, *p* = .50; intentions to wear a mask in crowded public places, *F*(1, 375) = 0.02, η_*p*_^2^ < 0.001, *p* = .89; or intentions to social distance in crowded public places, *F*(1, 375) = 1.43, η_*p*_^2^ = 0.004, *p* = .23 (see Table 4). As in Study 1, we also looked at the effect of the manipulation of identification with humanity on the overall composite of health-related behavioral intentions, which remained non-significant, omnibus *F*(1, 375) = 0.01, η_*p*_^2^ < 0.001, *p* = .93 (see Table 4).

#### Helping Behaviors

Regarding Hypothesis 2d, a model including the manipulation of identification with humanity and COVID-19 vaccination status predicted participants’ interest in learning how to help secure masks for others, omnibus χ^2^(4, *N* = 378) = 14.18, *p* < .01. However, this was only because vaccinated individuals were more likely to express interest in helping, *b* = 0.80, *p* < .001; the manipulation of identification with humanity did not independently affect interest in helping, *b* = 0.15, *p* = .49. Regarding Hypothesis 2e, the manipulation of identification with humanity (even after controlling for COVID-19 vaccination status) had no effect on participants’ effort to help secure rides to vaccination appointments, *F*(1, 375) = 0.65, η_*p*_^2^ = 0.002, *p* = .42 (see Table 4).

### Discussion

Consistent with preregistered hypotheses, the manipulation of identification with humanity marginally increased the full identification with all humanity scale, significantly increased bond with all humanity, but had no effect on concern for all humanity. Not supporting hypotheses, but consistent with the results of Study 1, identification with humanity had no causal effect on health-related COVID-19 behaviors or helping toward others.

## General Discussion

The present research took a social identity approach to health-related behaviors, focusing exclusively on identification at the highest level of abstraction—the human level (Turner et al., 1987). We reasoned that identification at this “human” level should be particularly relevant for mobilizing the kind of health-related behaviors (e.g., mask wearing, social distancing) that protect fellow humans from contracting COVID-19, a viral outbreak that has infected hundreds of millions of people across national and cultural boundaries. Thus, COVID-19 represents a collective threat (rather than a personal one), and identification with the human ingroup should promote behaviors that benefit and protect others from COVID-19. Because social identities are often contextually activated (e.g., see Turner et al., 1987; Oakes et al., 1994), the present research examined whether activating the human identity is an effective public-health strategy for improving health-related behaviors during COVID-19. In particular, this research examined whether the human identity can be situationally activated using an experimental manipulation (Goal 1), whether activating the human identity causally increases intentions to engage in behaviors that minimize the spread of COVID-19 (e.g., mask wearing, social distancing, receiving a COVID-19 “booster” vaccine; Goal 2), and whether activating the human identity causally increases helping behaviors that protect vulnerable communities from COVID-19 (Goal 3).

### The Effect of Identification With Humanity on Behavioral Intentions and Helping During COVID-19: Findings, Limitations, and Future Directions

Regarding Goals 2 and 3 of the research, results suggest that the manipulation used to activate the human identity had no causal effect on health-related COVID-19 behaviors—even after controlling for the progression of time, COVID-19 positivity rates (Study 1), and participants’ vaccination status (Study 2). The non-significant effect was consistent regardless of how the primary outcomes were measured—that is, whether outcomes were operationalized as behavioral *intentions* to wear a mask (Study 1 and 2), clean (Study 1), social distance (Study 1 and 2), or receive a COVID-19 “booster” vaccine in the future (Study 2); or whether outcomes were operationalized as *actual* behaviors to help secure masks (Study 1) or rides to vaccination appointments (Study 2) for vulnerable communities. The effect of the manipulation on COVID-19 behaviors also remained non-significant whether data were collected in a sample of college students from a university that held in-person classes during the pandemic (Study 1), or an online sample of U.S. adults that were comparatively more diverse in terms of race, gender, and age. Finally, the effect of the manipulation on COVID-19 behaviors remained non-significant despite roughly doubling the statistical power between studies, from approximately 100 participants per condition (Study 1) to 190 participants per condition (Study 2). One interpretation of these data is that the true size of the effect of activating the human identity on health-related COVID-19 behaviors is so small that the current studies were not sufficiently powered to detect it. Another possibility is that, even though individual differences in identification with all humanity have been found to be correlated with health-related COVID-19 behaviors (e.g., Barragan et al., 2021; Wang et al., 2021), situationally activating the human identity has no real causal effect on health-related behaviors. We cannot be entirely sure given the present studies alone.

However, given these consistent non-significant effects, it is possible that the current manipulation of identification with humanity was limited in its ability to cause changes in health-related COVID-19 behaviors. In other studies that experimentally manipulate identification with humanity and found causal effects on intergroup forgiveness (e.g., Wohl and Branscombe, 2005; Greenaway et al., 2011), the manipulation was contextualized around intergroup conflict and an explicit ingroup vs. outgroup contrast was salient. Thus, perhaps a manipulation of identification with humanity that contextualizes the COVID-19 pandemic and contrasts the human ingroup from the COVID-19 “outgroup” would have been more successful in causally affecting health-related COVID-19 behaviors (e.g., see Reese et al., 2020). However, previous research has shown a contrasting outgroup does not need to be salient to promote attitudes and behaviors that favor the ingroup (Gaertner et al., 2006). Indeed, in the only other study that successfully manipulated identification with humanity, Reese and colleagues’ (2015) subtle use of the same “globe” image from Study 1 increased donations to a global charity—even without contextualizing the manipulation or providing an ingroup–outgroup contrast. Instead, we believe that the current manipulation of identification with humanity did not have a causal effect on health-related behaviors and helping because the manipulation did not increase the appropriate factor of identification with all humanity. We discuss this possibility in greater detail in the following section.

While the current research did not show a causal effect of identification with humanity on health-related COVID-19 behaviors, it is important to weigh these results in light of other findings. Much research taking a social identity approach has demonstrated that social identification has both correlational and causal effects on personal, health-related behaviors (for reviews, see Haslam et al., 2009; Jetten et al., 2017), including in the context of COVID-19 (Van Bavel and Boggio, 2020). Additionally, new research consistently demonstrates an association between identification with broader, more inclusive groups (e.g., all of humanity, global citizens) and personal behaviors that benefit and protect fellow humans from COVID-19 (Barragan et al., 2021; Deng, 2021; Wang et al., 2021). Moreover, there is some preliminary support for theoretically grounded approaches that outline the unique social identification processes relevant for identification with humanity and COVID-19 (e.g., Reese et al., 2020). For instance, work by Zagefka (2021) suggests appraisals of the COVID-19 pandemic as a collective threat that requires global cooperation predicted stronger identification with all humanity, which in turn predicted stronger willingness to donate money to countries negatively affected by COVID-19. Thus, although the current research did not uncover a causal effect of identification with humanity on health-related COVID-19 behaviors, there remain promising avenues for future research. This includes, among others, examining the causal effects of superordinate social identification processes (e.g., ingroup identification, ingroup norms, perceived efficacy of ingroup goals) on appraisals of and health-related behavioral responses to viral pandemics.

### Manipulating Identification With Humanity: Findings and Future Directions

The results for Goal 1 of the current research were more encouraging. In our attempt to manipulate identification with humanity, participants in the human-identity condition focused on an image of our planet, and then reflected on and wrote about what it means to be a human being. Compared to the control condition, participants who had their human identity “activated” scored higher on a particular cluster of items from the identification with all humanity scale (McFarland et al., 2012). While initially McFarland et al. (2012) suggested the identification with all humanity scale represented a unidimensional construct, newer research indicates identification with all humanity is a higher-order construct with two subfactors (Reese et al., 2015; Reysen and Hackett, 2016; McFarland et al., 2019; Hamer et al., 2021). Researchers now recognize that the items representing *bond with all humanity* focus on cognitive categorization and affective feelings of closeness with the human ingroup (the identity component), and the items representing *concern for all humanity* focus on caring for and wanting to help the human ingroup (the behavioral component; see Hamer et al., 2021). Because the “bond” and “concern” factors have differential associations with attitudinal and behavioral outcomes (e.g., Reese et al., 2015; Reysen and Hackett, 2016; Sparkman and Hamer, 2020), research has increasingly examined them separately.

When examining results separately for the bond and concern subfactors in the present research, the manipulation of identification with humanity consistently increased (albeit with small effects, Cohen’s *d*s = 0.21 – 0.27) the bond with all humanity factor. This makes some sense given the writing prompt of the experimental condition, which asked participants to reflect on what it means to be a human being, including what they have in common with and how they are connected to humans all over the world. However, the manipulation of identification with humanity had no effect on items representing the concern for all humanity factor. Thus, separately analyzing the bond and concern factors demonstrates the current manipulation of identification with humanity increased the extent to which participants categorized themselves as, and felt emotionally close to, the human ingroup, but it did not increase participants’ concern for or desire to help the human ingroup.

These results suggest manipulations of identification with humanity can be quite nuanced, emphasizing one particular factor of the identity (e.g., psychological bond with the human ingroup) over another (e.g., behavioral concern for the human ingroup; see Reese et al., 2015). As such, the specific factor of identification with humanity being manipulated may have implications for different outcomes. For instance, manipulations of identification with humanity that focus on forming a psychological bond or connection to the human ingroup, such as the manipulation used in this research, may be more effective at improving *cognitive* and *affective* reactions toward the self and others. On the other hand, manipulations of the human identity that focus on caring for and recruiting the motivation to help the human ingroup may be more effective at promoting *behaviors* that benefit all humans. For this reason, we believe the current research did not show a causal effect on health-related behaviors, or helping protect others from COVID-19, because the manipulation of identification with humanity increased the cognitive-affective component (*bond*) when it should have increased the behavioral component (*concern*). Future research would do well to develop a manipulation that successfully activates concern for all humanity, and then examine whether this manipulation causally affects health-related behaviors that can protect the self and others from COVID-19 (or future viral outbreaks).

Our manipulation of identification with humanity may also act similarly to manipulations identified in the common ingroup identity model (CIIM; for reviews, see Gaertner and Dovidio, 2000). From this perspective, activating a common, “human” identity recategorizes all outgroups to be part of the same inclusive ingroup, and this, in turn, redirects the power of ingroup biases to promote more positive outcomes toward “former” outgroups. Keeping in mind the distinction between the bond and concern factors of identification with humanity, future research could investigate whether manipulations that activate a psychological bond with the human ingroup are more effective at reducing stereotyping (a cognitive outcome) and prejudice (an affective outcome) toward different outgroups, and also whether manipulations that activate the desire to help the human ingroup are more effective at reducing discrimination or violence toward outgroups (both of which are behavioral outcomes). Given how little research has attempted to experimentally manipulate the human identity, and also how difficult it is to causally increase inclusive social identities in general (Reysen et al., 2021), testing the effectiveness of different manipulations of the human identity on different affective, cognitive, and behavioral outcomes is a fruitful area for future research.

Overall, we believe the results reported here suggest our manipulation of identification with humanity can be successfully used to activate one’s psychological bond with the human ingroup (but not necessarily their concern for or desire to help the human ingroup). As such, we encourage researchers interested in this inclusive social identity to use and build upon this manipulation of identification with humanity. Given the preliminary nature of this manipulation, however, more research is needed to replicate the effects, increase the strength of the manipulation, and assess its reliability in different contexts and cultures. This is especially relevant considering the samples in the current research are from the United States and therefore likely to be higher in individualistic (rather than collectivist) orientations, which itself has implications for health-related behaviors during COVID-19 (e.g., see Lu et al., 2021).

### Benefits of the Current Research to Vulnerable Communities During COVID-19

As part of their involvement in the current research, participants had the opportunity to complete tasks with real financial benefits toward organizations that help communities especially vulnerable to the COVID-19 pandemic. In Study 1, participants had the opportunity to complete tasks that donated money to the #MaskUpMKE fund of the United Way of Greater Milwaukee and Waukesha County, an initiative to make and distribute face masks to essential workers, communities of color, and other individuals in the region. In Study 2, participants had the opportunity to complete tasks that donated money to the Vaccine Access Fund, an initiative providing free rides to vaccine sites for Black and Latino adults, people living on low incomes, and others for whom transportation is a barrier. In total, participants’ efforts generated $113.70 to these communities, a donation that was paid for by the researchers. Although we acknowledge that this component of the research is unlikely to fully remedy long-standing issues of inequity and injustice, it is an approach we took to ensure that this research—at least in part—benefited the communities and/or ideas studied.

## Conclusion

Our research took a social identity approach to the current COVID-19 pandemic and examined whether situationally activating the most inclusive, “human” identity could be used as an effective public-health strategy to promote personal, health-related behaviors that reduce the spread of COVID-19. Across two studies, our findings consistently suggest that the manipulation of identification with humanity did not have any causal effect on health-related behavioral intentions (e.g., to wear a mask, social distance, etc.) or helping behaviors that protect others from COVID-19. However, the results do show that the manipulation of identification with humanity consistently increased participants’ psychological bond with the human ingroup (but not their concern for the human ingroup). We believe such experimental manipulations that seek to activate our collective, “human” identity may be used to address a range of global issues that affect all human beings—not only viral pandemics, but climate change, refugee crises, international conflict, and possibly other crises. As astronaut Scott Kelly mentioned at the start of the COVID-19 pandemic, “All people are inescapably interconnected, and the more we can come together to solve our problems, the better off we will all be.”

## Data Availability Statement

The datasets presented in this study can be found in online repositories. The names of the repository/repositories and accession number(s) can be found below: https://osf.io/2pes6/ at the Open Science Framework (OSF).

## Ethics Statement

The studies involving human participants were reviewed and approved by the University of Wisconsin–Eau Claire Institutional Review Board (IRB) Committee. The patients/participants provided their informed consent to participate in this study.

## Author Contributions

DS contributed to conception and design of all studies, receiving Institutional Review Board approval, statistical analysis, and wrote the manuscript. KK and EN contributed to conception, design, Institutional Review Board approval, and statistical analysis of Study 1. All authors approved the submitted version of the article.

## Conflict of Interest

The authors declare that the research was conducted in the absence of any commercial or financial relationships that could be construed as a potential conflict of interest.

## Publisher’s Note

All claims expressed in this article are solely those of the authors and do not necessarily represent those of their affiliated organizations, or those of the publisher, the editors and the reviewers. Any product that may be evaluated in this article, or claim that may be made by its manufacturer, is not guaranteed or endorsed by the publisher.

## Footnotes

1
https://osf.io/qzmhs/?view_only=f24f5c96406b49948227eebf327941b7

2
https://osf.io/phm7u/?view_only=1ca9d445be674e30ada93912fde4a895

3
https://osf.io/2pes6/?view_only=84b51dfcdb9347a08788426c433e5d28

4 Due to researcher error, a single question asking participants to report their political ideology was not removed from our standard demographics page before launching both studies. This was the only other measure collected in our studies that was not preregistered. We have not conducted any analyses on this measure and therefore do not report them here.

5 The target group “humans all over the world” was used in place of the original phrasing of “people all over the world” to specifically highlight the human identity. However, previous research suggests the concepts of “humans” and “people” have similar prototypical meanings (Carmona et al., 2020).

6 Data collection was stopped once the Prolific survey platform indicated 400 participants opted into the survey. However, some Prolific users often choose to return their submission before completing the survey, and 11 participants did so in this case. Once data collection is stopped on Prolific, it cannot be easily restarted; a new survey page must be generated to recruit additional participants. Thus, we chose to proceed with data analysis with a sample of 389 participants.

7 Participants who took longer received a bonus payment of up to $0.75.
